# Department of Agriculture Reports

**Published:** 1897-09

**Authors:** 


Department of Agriculture Reports. The Twelfth and Thirteenth Annual Reports of the Bureau of Animal Industry.

This volume contains so much of interest to the veterinary profession, so much of importance to the welfare of the live-stock interests of our country, that we had hoped ere this to have had it more thoroughly reviewed by one of the staff contributors to the Journal.

A glance at its contents will give at once an impressive idea of the great scope of work of the Bureau, and its well-trained experts afford a valuable and complete review of the existing contagious and infectious diseases of live-stock during the past two years, with a *résumé* of the progress made in their better control and eradication. The live-stock interests of our country have had much in the past to thank the Bureau of Animal Industry for, and the real value of its investigations and work grow more forceful each year. To this department, which gives so large a return of real value to those of our people engaged in agriculture, there should be the freest helping-hand given to enlarge and make more effective the benefits to be derived from this work, which appeals so directly to the welfare of 55 per cent. of our people and indirectly to the entire 73,000,000 of people within our borders.

